# Elevated D-Dimers and Right Ventricular Dysfunction on Echocardiography for Diagnosis of Pulmonary Embolism: A Validation Study

**DOI:** 10.7759/cureus.10778

**Published:** 2020-10-03

**Authors:** Hamid Sharif Khan, Asim Javed, Muhammad Mohsin, Shabana Kousar, Summaya Salik Malik, Jahanzeb Malik

**Affiliations:** 1 Cardiology, Rawalpindi Institute of Cardiology, Rawalpindi, PAK

**Keywords:** acute pulmonary embolism, d-dimers, right ventricular dysfunction, rv strain, pulmonary infarction

## Abstract

Background

There is an increasing need to explore other non-invasive techniques for the diagnosis of pulmonary embolism in resource-limited countries.

Objective

To assess the validity of elevated D-dimer levels and right ventricular (RV) dysfunction on echocardiography in predicting definite massive pulmonary embolism among patients diagnosed with massive pulmonary embolism using computed tomography (CT) pulmonary angiography as the gold standard.

Methods

The patients with acute massive pulmonary embolism on CT pulmonary angiography were included. The participants underwent 12-lead electrocardiography, assessment of D-dimer levels, and bedside echocardiography to determine right ventricular dysfunction. The data were recorded on a proforma and analyzed using IBM SPSS software version 26.0 (IBM Corp., Armonk, NY).

Results

There were 160 patients in the study. The mean age was 49.19 ± 14.89 years. Elevated D-dimer levels were seen in 80.60% of the patients whereas ventricular dysfunction on echocardiography was seen in 90.00% of the patients. The sensitivity and specificity of elevated D dimer levels were 78.99% and 14.60%, respectively. The positive predictive values (PPV) and negative predictive values (NPV) for elevated D-dimer levels were 72.87% and 19.35%, respectively. In contrast, the sensitivity of ventricular dysfunction was 94.96% and specificity 24.39%. PPV was found to be 78.47% and NPV was 62.50%.

Conclusion

Positive D-dimer levels and ventricular dysfunction on echocardiography are sensitive enough to consider the diagnosis of massive pulmonary embolism but lack adequate specificity, thus, necessitating the presence of other noninvasive tests.

## Introduction

Acute pulmonary embolism (PE) is a clinical entity that is associated with increased rates of morbidity and mortality with most deaths occurring within a few hours after presentation [1]. The disease is characterized by a blood clot or thrombus obstructing the pulmonary arterial vasculature causing reduced blood flow to the lungs which in turn leads to reduced tissue perfusion, leading to organ dysfunction thus increasing risks of mortality [2,3]. Early diagnosis and prompt management is of paramount importance in reducing the mortality associated with acute massive PE [4].

Clinical parameters that could lead to the diagnosis of acute massive PE are unreliable and misdiagnosis can have serious consequences [5]. An incorrect diagnosis can expose the patient to unnecessary anticoagulation. Considering these issues, a test or combination of tests need to be established.

Different tests have been used to help in establishing a differential diagnosis of acute massive PE. Among these tests, computed tomography (CT) pulmonary angiogram (CTPA) has high validity in diagnosing acute massive pulmonary embolism (thrombus involving the main pulmonary artery with CT obstruction index of more than 50%) along with right ventricular dilatation with right to left ventricular ratio of greater than 0.90 [6,7]. Unfortunately, CTPA is not available in most primary and secondary centers of the country and hence there requires certain laboratory tests or echocardiographic parameters to help in diagnosing massive PE. Elevated D-dimer levels and right ventricular dysfunction on echocardiography are two potential noninvasive tests that could be done to diagnose acute PE. D-dimer is formed following the lysis of cross-linked fibrin by plasmin and is hence elevated in massive pulmonary embolism. It can also be elevated in other conditions, but, a negative D-dimer level can be used to rule out PE [8,9].

Echocardiography, transthoracic, or transesophageal is a valuable noninvasive tool in diagnosing acute massive pulmonary embolism [10]. It aids indirect visualization of thrombus in the main pulmonary artery or the right-sided cardiac chambers; right atrium (RA) and right ventricle (RV) [11]. The presence of thrombus in the pulmonary arteries can be indirectly confirmed due to unexplained dilatation or dysfunction of right ventricular with or without tricuspid valve insufficiency [12]. These latter parameters exhibit a sensitivity of about 50% and a specificity of about 90% for pulmonary embolism in various studies [13]. Compared to transthoracic echocardiography, transesophageal echocardiography with its improved resolution can visualize thrombi in the central pulmonary arteries (main, right, proximal portion of left) with high specificity (>90%), but its sensitivity has not been evaluated in unselected patients with pulmonary embolism [14].

## Materials and methods

The prospective validation study was conducted at Rawalpindi Institute of Cardiology Rawalpindi from January 2019 to December 2019 following approval from the ethical review committee. All patients had given their informed consent before the commencement of the study. The sample size was calculated using software with an expected population of 271 patients, an anticipated frequency of 0.50, confidence intervals of 95%, and a design effect of 1.0. There were 160 patients with confirmed acute massive pulmonary embolism on CT pulmonary angiography (characterized by filling defect due to thrombus in the main pulmonary artery or in one of its branches) who had been included after randomization. The patients underwent 12-lead electrocardiography (ECG) using Mortara Instrument ELI 250 (Welch Allyn® USA).

D-Dimer qualitative assessment was done in the hematology laboratory. Commercially prepared reagent ‘TechnoLEIA® CTM D-Dimer Latex Kit’ (Diapharma Group, Inc., West Chester, OH, USA) was used for analysis. CTM D-Dimer latex test is intended for the rapid qualitative or semi-quantitative evaluation of circulating derivatives of cross-linked fibrin degradation products (XL-FDP) in human plasma. CTM D-Dimer latex is a rapid agglutination assay utilizing latex beads coupled with a highly specific D-Dimer monoclonal antibody. Undiluted plasma D-Dimer concentration was regarded as positive or negative depending on plasma value, either greater or less than 200 ng/ml, respectively [15].

Transthoracic echocardiography (TTE) was done using Aplio 300 CV ultrasound systems to look for right ventricular dysfunction. The criteria used for diagnosing this dysfunction included ratio of right ventricle to left ventricle (RV/LV), end-diastolic diameter greater than 1 in apical 4 chamber view, right ventricular end-diastolic diameter (RVEDD) greater than 30 mm, and paradoxical right ventricular septal motion [11]. In presence of thrombus in major pulmonary arteries, the right ventricular free wall shows an overall reduced contraction with sparing of the apex. This phenomenon is called a McConnel sign [16].

CT pulmonary angiography was done to confirm the diagnosis. Criteria for massive pulmonary embolism were defined as embolization of the pulmonary artery with an obstruction index of greater than 50% along with right ventricular dilatation with an RV/LV ratio greater than 0.90 [6,7].

The data was collected on a pre-tested questionnaire by the investigator after taking informed consent from the patient. Each participant was allotted an identification number and the confidentiality of the participant was safeguarded. The data was analyzed using IBM Statistical Package for Social Sciences (SPSS), version 26.0 (IBM Corp., Armonk, NY). Mean and standard deviation were calculated for quantitative variables such as age. Descriptive statistics were calculated for qualitative variables. A 2 x 2 table was constructed to calculate sensitivity, specificity, positive predictive value (PPV), and negative predictive value (NPV) for D-dimer levels, right ventricular dysfunction, and combination of both with CTPA as the standard. Regression analysis was done to control confounding.

## Results

There were 160 participants in the study. The mean age of the participants was 49.19 ± 14.89 years. There were 100 male (62.50%) and 60 female (37.50%) participants in the study. The mean heart rate was 112 ± 8 per minute. There were 123 participants (76.90%) with a systolic blood pressure greater than 100 mmHg, D-dimer was positive in 129 participants (80.60%). The electrocardiogram findings included sinus rhythm in 15 participants (9.38%), sinus tachycardia in 111 participants (69.38%), and an S1Q3T3 pattern in 34 participants (21.25%). The echocardiography showed right ventricular dysfunction in 144 participants (90.00%). Hemodynamic instability was present in 123 participants (76.90%). Regression analysis was not significant for confounders such as age and comorbid conditions.

A 2 x 2 table was constructed to determine the validity of the D-dimer test. It is shown in Table 1.

**Table 1:** A 2 x 2 table for D-dimer test

D-dimer test	CT pulmonary angiography positive for pulmonary embolism	CT pulmonary angiography negative for pulmonary embolism	Total
Positive D-dimer test	94	35	129
Negative D-dimer test	25	6	31
Total	119	41	160

As presented in Table 1, elevated D-dimer exhibited sensitivity of 78.99% (confidence interval, CI: 70.57% to 85.92%) for diagnosing acute massive pulmonary embolism, but lacked specificity (14.60%; CI: 5.57% to 29.17%). The overall accuracy was 62.50% (CI: 54.51% to 70.02%). The positive predictive value was 72.87% (CI: 69.66% to 75.86%). The negative predictive value of elevated D-dimer was low and found to be 19.35% (CI: 9.58% to 35.21%).

Another 2 x 2 table was constructed to determine the validity of right ventricular dysfunction. It is shown in Table 2.

**Table 2:** A 2 x 2 table for right ventricular dysfunction

Echocardiography with right ventricular dysfunction	CT pulmonary angiography positive for pulmonary embolism	CT pulmonary angiography positive for pulmonary embolism	Total
Present	113	31	144
Absent	6	10	16
Total	119	41	160

As per the calculations in Table 2, right ventricular dysfunction on echocardiography exhibited a high sensitivity level (94.96%; CI: 89.35% to 98.13%) for diagnosing acute pulmonary embolism. However, like elevated D-dimer levels, its specificity was low (24.39%; CI: 12.36% to 40.30%). Its overall accuracy was slightly higher than the elevated D-dimer levels at 76.88% (CI: 69.56% to 83.17%). The positive predictive value was comparable to elevated D-dimer levels (78.47%; CI: 75.30% to 81.34%).

## Discussion

Massive pulmonary embolism is a clinical entity associated with high rates of morbidity and mortality. Different tests have been used over the years for diagnosing and excluding massive PE so as to diagnose the disease early for early management. A single noninvasive test has not been independently sensitive and specific enough to consider the possibility of massive PE. CT pulmonary angiography with a filling defect in the main or pulmonary artery can be considered as a potential test. In a developing country like Pakistan, it is not readily available in most hospitals making it difficult to diagnose acute PE in cases of high clinical suspicion. Other non-invasive tests in such situations include elevated D-dimer levels and right ventricular dysfunction on transthoracic echocardiography.

In our study, positive D-dimer levels had high sensitivity (78.99%) increasing their value in ruling out the diagnosis of pulmonary embolism. However, the test had a low specificity (14.60%) reducing its usefulness as a stand-alone test. Similar results were seen in another study that found that positive D-dimer levels were associated with high sensitivity but low specificity [17]. The main reason being elevation in D-dimer levels can also be in conditions like advanced age, cancer, and sepsis [18]. In addition, our study showed a higher positive predictive value of 72.87% which was similar to a study showing a PPV of 79% [19]. Another study noted a PPV close to 100%, but in that case, elevated D-dimer assay was compared with ventilation/perfusion (V/Q) scan as opposed to CT pulmonary angiography [20].

Right ventricular dysfunction on echocardiography secondary to thrombus in the main pulmonary artery or the right and left pulmonary arteries is potentially considered as a reliable test for diagnosing massive PE with high validity as concluded by a study that showed sensitivity of 50% and specificity of 90% in diagnosing massive PE [21]. In our study, it showed a high sensitivity of 94.96% in diagnosing massive PE but had a low level of specificity at 24.39%. The main reason for the low specificity is unknown but appears to be secondary to certain pathologies of lungs that could lead to right ventricular hypertrophy like chronic obstructive pulmonary disease or pulmonary hypertension.

The right ventricular dysfunction on echocardiogram has an important role for prognosis as seen in different studies that show a correlation between adverse outcomes and right ventricular dysfunction in pulmonary embolism [22]. Although in our study we did not study the correlation of RV dysfunction with adverse outcomes, it can however be used to risk-stratify patients with acute PE [23].

There are a few limitations to the study. The sample size of the study is small and the study was limited to one center. Our study is the first of its kind in the country assessing the validity of certain tests in diagnosing massive pulmonary embolism. We found that the two noninvasive tests, elevated D-dimer levels and right ventricular dysfunction pattern on echocardiography, have high levels of sensitivity to rule in acute massive PE but lack specificity to rule out massive PE. These tests can be considered for diagnosing massive PE if the clinical probability of massive PE is high, however, they are not reliable to rule out massive PE completely and other (noninvasive) tests like CT pulmonary angiography and/or ventilation-perfusion scan may be needed to rule out massive pulmonary embolism.

## Conclusions

Elevated D-dimer levels and RV dysfunction on echocardiography are sensitive enough to rule in the diagnosis of massive pulmonary embolism but lack specificity to rule out massive PE, thus necessitating the presence of other noninvasive tests (V/Q scan, CTPA) to rule out acute PE.
